# “*In starvation, a bone can also be meat*”: a mixed methods evaluation of factors associated with discarding of long-lasting insecticidal nets in Bagamoyo, Tanzania

**DOI:** 10.1186/s12936-022-04126-5

**Published:** 2022-03-24

**Authors:** Edith P. Madumla, Sarah J. Moore, Jason Moore, Emmanuel Mbuba, Edgar M. Mbeyela, Ummi A. Kibondo, Selemani C, Mmbaga, Dickson Kobe, Jitihada Baraka, Daniel Msellemu, Johnson K. Swai, Zawadi M. Mboma, Olukayode G. Odufuwa

**Affiliations:** 1 Vector Control Product Testing Unit, Environmental Health and Ecological Science Department, Ifakara Health Institute, Bagamoyo, Tanzania; 2 Nelson Mandela African Institution of Science and Technology, Tengeru Arusha, Tanzania; 3 Vector Biology Unit, Epidemiology and Public Health Department, Swiss Tropical and Public Health Institute, Kreuzstrasse 2, Allschwil, 4123 Basel, Switzerland; 4 University of Basel, St. Petersplatz 1, CH-4002 Basel, Switzerland; 5 London School of Hygiene and Tropical Medicine, Keppel Street, London, WC1E 7HT UK

**Keywords:** Focus group discussions, Mixed methods, Bed net, Long-lasting insecticidal nets, Too torn, Malaria, Discarding, Mosquitoes, Tanzania

## Abstract

**Background:**

Between 2000 and 2019, more than 1.8 billion long-lasting insecticidal nets (LLINs) were distributed in Africa. While the insecticidal durability of LLINs is around 3 years, nets are commonly discarded 2 years post distribution. This study investigated the factors associated with the decision of users to discard LLINs.

**Methods:**

A mixed-method sequential explanatory approach using a structured questionnaire followed by focus group discussions (FGDs) to collect information on experiences, views, reasons, how and when LLINs are discarded. Out of 6,526 households that responded to the questionnaire of LLINs durability trial, 160 households were randomly selected from the households in four villages in Bagamoyo Tanzania for FGDs but only 155 households participated in the FGDs. Five of the household representatives couldn’t participate due to unexpected circumstances. A total of sixteen FGDs each comprising of 8–10 adults were conducted; older women (40–60 years), older men (40–60 years), younger women (18–39 years), younger men (18–39 years). During the FGDs, participants visually inspected seven samples of LLINs that were “too-torn” based on Proportionate Hole Index recommended by the World Health Organization (WHO) guidelines on LLIN testing, the nets were brought to the discussion and participants had to determine if such LLINs were to be kept or discarded. The study assessed responses from the same participants that attended FGD and also responded to the structured questionnaire, 117 participants fulfilled the criteria, thus data from only 117 participants are analysed in this study.

**Results:**

In FGDs, integrity of LLIN influenced the decision to discard or keep a net. Those of older age, women, and householders with lower income were more likely to classify a WHO “too-torn” net as “good”. The common methods used to discard LLINs were burning and burying. The findings were seen in the quantitative analysis. For every additional hole, the odds of discarding a WHO “too-torn” LLIN increased [OR = 1.05 (95%CI (1.04–1.07)), p < 0.001]. Younger age group [OR = 4.97 (95%CI (3.25–7.32)), p < 0.001], male-headed households [OR = 6.85 (95%CI (4.44 –10.59)), p < 0.001], and wealthy households [OR = 3.88 (95%CI (2.33–6.46)), p < 0.001] were more likely to discard LLINs.

**Conclusion:**

Integrity of LLIN was the main determinant for discarding or keeping LLINs and the decision to discard the net is associated with socioeconomic status of the household, and the age and gender of respondents. WHO “too torn” nets are encouraged to be used instead of none until replacement, and disposal of nets should be based on recommendation.

**Supplementary Information:**

The online version contains supplementary material available at 10.1186/s12936-022-04126-5.

## Background

Between 2000 and 2019, 67% of the reduction in malaria mortality was attributed to the scale up of long-lasting insecticidal nets (LLINs) and behaviour change campaigns (BCC), which promoted their use [1, 2]. It is estimated that more than 1.8 billion LLINs were distributed in Africa between 2000 and 2019, mainly through mass distribution campaigns [2]. In Tanzania, various distribution programmes such as The School Net Programme, the Universal Coverage Campaign (UCC) and Antenatal Care Unit (ANC) distributed millions of nets [1, 3]. Unfortunately, evidence compiled in recent years on the durability studies of LLINs found a median survival of 2 years and only 50% of nets remain in use until the next campaign [4, 5].

The studies to assess LLIN durability are often called community trial “Phase 3” of insecticidal nets, in which nets are evaluated to assess their loss (attrition), effect on mosquitoes (bioefficacy), retainment of insecticides (insecticide residual), damages (fabric integrity/net integrity which refers to the survivorship and ability of a bed net to maintain its insecticidal and physical condition for a longer time) and acceptance after three years of field use [6, 7]. The damages of LLINs are determined based on proportionate hole index (pHI), which refers to the composite measure of the holes from four-hole size categories in centimetre: 0.5–2, 2–10, 10–25 and > 25, practically measured using smaller than a thumb, larger than a thumb but smaller than a fist, larger than a fist but smaller than a head and larger than a head, respectively. Based on these sizes of holes found on a net, the pHI value is estimated and then divided into three categories: a bed net of a total hole surface area of < 0.001m^2^ (pHI < 64) is considered as “good”, a bed net of a total surface of ≤ 0.1m^2^ (pHI ≤ 642) is considered “damaged” and a torn bed net of a total surface area of > 0.1 m ^2^(pHI > 642) is considered as “too torn” to provide physical protection against mosquito bites [6, 8, 9]. Based on these studies, the World Health Organization (WHO) usually list or recommends LLINS that remain adequately insecticidal after three years of deployment, for this reason, mass campaigns of nets are implemented every 3 years, although most LLINs happen to be lost by the people once the nets are considered “too torn” – having many or large holes before 3 years.

Functional life of a LLIN is the amount of time that the bed net may be in service before it is rendered unusable due to changes in functional requirements like failure to repel or kill mosquitoes or get easily torn. The gap between the median functional life of a LLIN and mass distribution of LLINs has contributed to low population access to LLINs. Other reasons that affect access to LLINs include a low quality of LLIN, limited funding, reduction in the supply of LLIN due to financial difficulties, poor socioeconomic status, unequal access as well as poor infrastructures [5, 10–12]. Thus, population access to LLINs in sub-Saharan Africa, including Tanzania remains around 50% despite substantial efforts to increase global access [2].

It has been documented in Tanzania, that about 84% of LLINs distributed from different campaigns are discarded before the next campaign [5, 10], and campaigns happens at an interval of every 3–5 years. Individuals stop using mosquito nets [13–15], and discard them when they become extremely damaged as users no longer consider them to be protective [16–25]. However, a study conducted on durability of LLINs in Tanzania demonstrated that damaged LLINs were still insecticidal durable, that is, being able to repel and kill mosquitoes [26]. Therefore, it is of concern that there is widespread discarding of potentially protective nets especially given the low population access to LLINs.

The recommendation provided by the WHO on discarding bed nets acclaims that; LLIN should not be discarded in any water body because the residual insecticide on the net can be toxic to aquatic organism, especially fish [27]. They also recommended that old LLINs should be collected and the best option for disposal is a high-temperature incineration. [27]. They should not be burned in an open air. When these options are not possible, the recommended method of disposal is burial and burial should be away from water sources and preferably in non-permeable soil [27]. However, these recommendations are not followed by the communities, which may result into careless handling and discarding of insecticidal nets in the environment including burning LLINs in an open air. This may lead to the release of dioxins, which is harmful to human health. Improper burial on unspecified places can be toxic to aquatic organisms and a source of insecticide resistance. A recent study conducted in Kenya [23] described that insecticidal nets are washed in open water, repurpose for football posts, shopping bags and building in addition to being discarded either in the trash or burning. The behaviour of improper discarding of LLINs exposes the environment to contamination. The factors associated with discard of insecticidal nets in Tanzania have not been extensively researched. Thus, this study was conducted to understand factors associated with discarding of LLINs that could still be beneficial from a public health perspective [25].

## Methods

### Study area

**Fig. 1:**
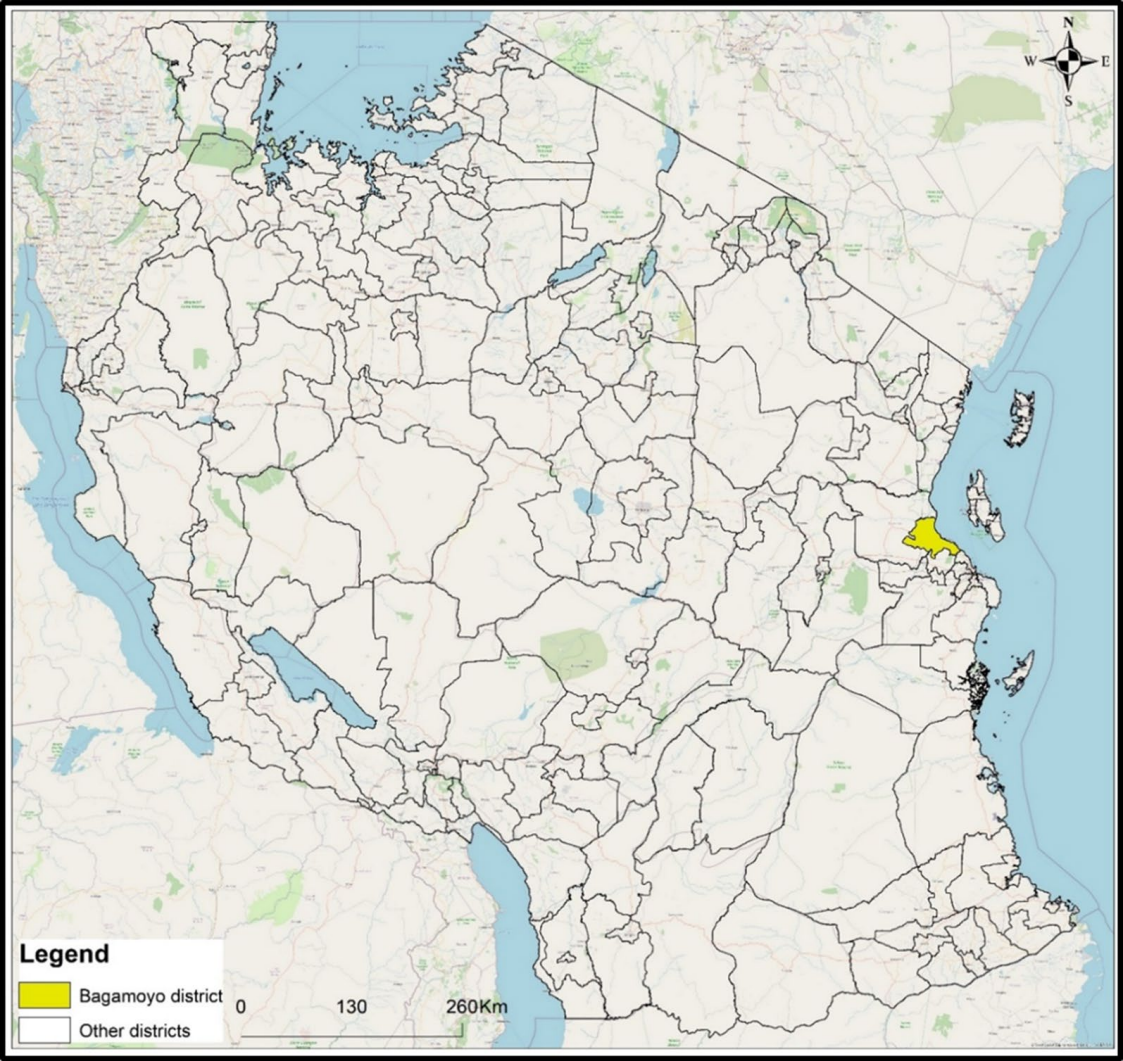
Map showing the location of Bagamoyo district in Tanzania where the study was carried out

The study was conducted in Bagamoyo district (Fig. 1), 70 km north of Dar es Salaam city, the economic hub of Tanzania. The population of Bagamoyo is approximately 311,740 people: 154,198 males and 157,542 females according to the 2012 Tanzania national census with an average household size of 4.4 [28]. More than 70% of the residents have primary education or higher [29]. Annual temperature ranges from 22–33 °C with rainfall between 800 and 1200 mm per year and mean relative humidity of 73%. Short rains (*vuli*) usually fall from November to December while long rains (*masika*) usually fall between March and May [30]. The driest months are June to September. The main economic activities in the area are small-scale farming of pineapples, maize, cassava, fishing, livestock keeping, mariculture (sea weed and prawn farming), salt production, trade and tourism [30].

### Study design

The study was a mixed-method sequential explanatory design [31], embedded in a bed net durability trial of five brands of LLINs in six villages in Bagamoyo namely Kiwangwa, Bago, Mwavi, Msinune, Mwetemo and Masuguru. Quantitative analysis data led to generation of themes for qualitative study. The FGD sessions captured information on participants’ experience, opinion and views regarding the factors associated with the discarding of insecticidal nets, their perception of bed net use and net care; and the causes of damage to nets in their communities.

The integration between quantitative and qualitative phases was connected during the middle stage in the research process. Participants who responded to the survey and reported to discard nets were the ones who met the criteria to attend FGD and thus were selected to participate in the FGD. Data collected during the quantitative survey informed the topic guide which was used to collect qualitative data.

**Fig. 2:**
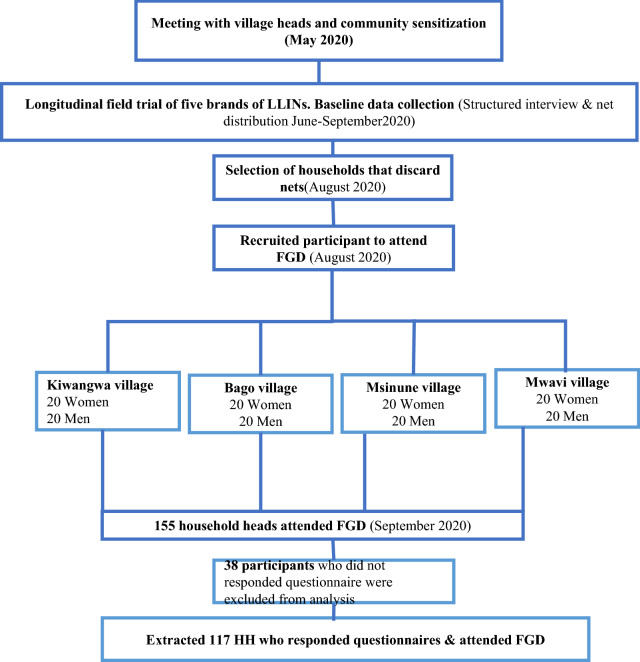
Flow chart of the study design

The participants were above 18 years of age and consented to participate in the study. These participants were put into four groups composed on similar characteristics which were age and gender. Each group had eight-to-ten participants seated in a circle with the moderator in the middle, while the note taker sat out of the circle but in a position where it was possible to see all the participants. The first group was of younger women aged 18–39, the second group was of older women aged 40–60, the third group made of younger men aged 18–39, and the last group was of older men aged 40–60 (Fig. 2). The groups were made such that participants could freely talk and discuss issues among their peers. This usually makes them comfortable when sharing their experiences.

**Fig. 3:**
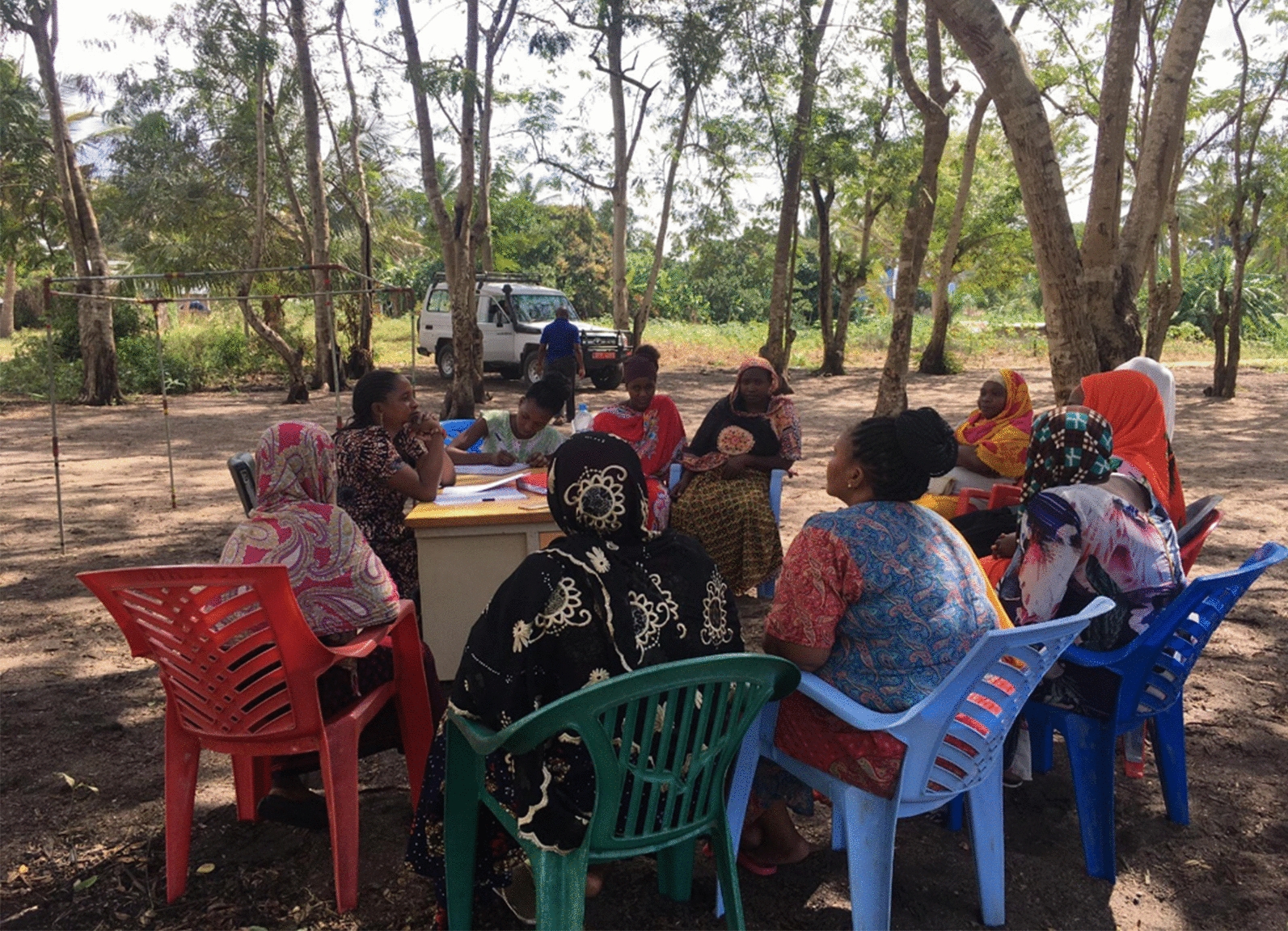
One of the focus group discussion sessions with younger women

A topic guide was used as an aid for smooth discussion including probing and rephrasing of words to elicit more information. A digital recorder was used to capture all the information communicated. Each discussion lasted for one hour. The discussions were conducted in Kiswahili, the locally spoken language (Fig. 3).

**Fig. 4:**
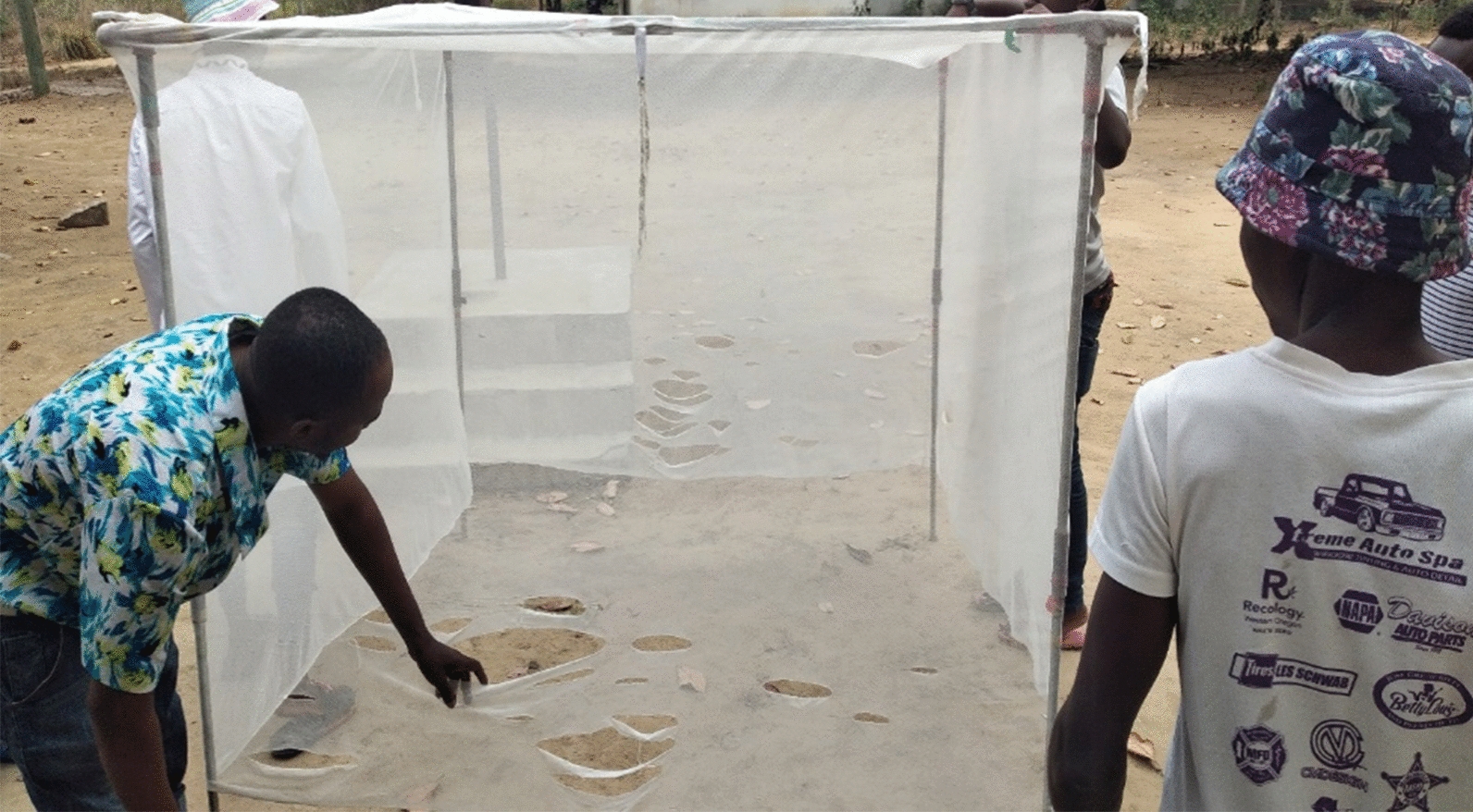
Younger men visually inspecting too torn nets to decide if they will keep the net or discard it

At the end of each FGD, seven 6 × 5 white LLINs from a previous LLIN durability trial [21] representing “too torn” nets based on WHO pHI were brought and presented to the FGD participants to inspect and decide if they would discard or keep the LLINs based on their visual (Fig. 4). The characteristics of the LLINs assessed by the FGD participants are shown (Table 1). The characteristics of the net such as cleaning practices (dirty vs clean) and materials (rough vs smooth) were considered to assess if they are associated with discarding of WHO “too torn” nets in Bagamoyo villages.

**Table 1:** Characteristics of the damaged nets assessed by the FGDs participants

Net ID	Net material	Net cleanliness	Number of holes	Proportionate hole index	WHO size	No of holes	Hole location
1	Rough	Clean	21	1,620	1	5	Mix
					2	11	
					3	4	
					4	1	
2	Rough	Clean	27	1,854	1	18	Mix
					2	4	
					3	3	
					4	2	
3	Rough	Clean	14	1,632	1	5	Mix
					2	3	
					3	5	
					4	1	
4	Rough	Clean	41	2,486	1	18	Mix
					2	14	
					3	8	
					4	1	
5	Rough	Dirty	22	816	1	19	Mix
					2	1	
					3	1	
					4	1	
6	Smooth	Dirty	79	6,142	1	38	Mix
					2	20	
					3	17	
					4	4	
7	Smooth	Dirty	133	4,987	1	87	Mix
					2	26	
					3	19	
					4	1	

Size 1: smaller than a thumb (0.5–2 cm), Size 2: larger than a thumb but smaller than a fist (2–10 cm), Size 3: larger than a fist but smaller than a head (10–25 cm) and Size 4: larger than a head (> 25 cm)

To quantitatively assess participants perception of the nets, holes in the seven LLINs were categorized into four groups: Size 1: smaller than a thumb (0.5–2 cm), Size 2: larger than a thumb but smaller than a fist (2–10 cm), Size 3: larger than a fist but smaller than a head (10–25 cm) and Size 4: larger than a head (> 25 cm), as per WHO recommendation [6]. The proportionate hole index (pHI) of each net was calculated [8], and all were above 642, thus “too torn” nets, indicating that they provide little physical protection against mosquito bites compared to intact ones.

### Factors associated with the discarding of LLINs using structured questionnaire

Information was primarily collected from the head of households. The data extracted from the durability baseline questionnaire includes age, sex, level of education, number of occupants per household, house structure, livestock, assets owned, source of light and occupation. Other data extracted were availability of nets, their usage patterns, perception of bed nets, net care attitude questions around net care and repair as well as reasons for discarding nets, including how, when and where the LLINs are discarded. A net attitude score was also estimated to assess attitudes toward the net care and repair practices [32] using questions on perception of bed nets and net attitude provided as Additional file 1: Tables S1 and S2, respectively.

### Sample size

The quantitative data from LLIN durability trial was used to obtain qualitative members for FGD. A structured questionnaire from the trial collected demographic and socioeconomic information from 6,526 households from six villages in Bagamoyo District (Mbuba et al. Unpublished). One hundred and sixty households among them who reported discarding of nets were selected randomly to participate in the FGDs from four villages, which were Kiwangwa, Bago, Mwavi and Msinune. Each village contributed 40 households and an individual represented each household in the FGD. A total number of four FGDs were held per village, thus sixteen FGDs were conducted in this study. Findings from the FGDs guided extraction of the information of 117 participants who also responded to the structured questionnaire during the baseline survey of the net durability trial.

### Data analysis

Data collected during the quantitative LLIN durability trial informed the topic guide which was used to collect qualitative data [33]. About 117 FGD households were found to match with the quantitative data and were selected for qualitative analysis. The audiotapes from the FGD recording were transcribed verbatim independently by two researchers and checked for completeness. Transcripts were then entered into the NVivo software [34, 35] and codes were developed thematically. Later selected quotes were translated into English. Thematic framework approach was used for analysis according to Ritchie et al. [33]. The analysis was conducted based into six stages which are: 1) Familiarization of data, 2) Coding, 3) Searching for themes, 4) Reviewing themes, 5) Defining and naming themes and lastly 6) writing up [36–38]. After initial coding of all transcripts, the next step was to look for similarities and differences between patterns and themes. Relationships and connections between themes were established and the final step was the interpretation of data.

For quantitative data analysis, STATA 16 statistical package software was used [39]. Variables; household socioeconomic status (SES) and positive net attitude were derived from a weighted score in a principal component analysis (PCA) [40]. Variables used in the PCA analysis were categorized into binary. For SES, variables were categorized on have vs have not or modern vs traditional; For positive net attitude, variables were categorized based on definitely could and probably could vs definitely could not and probably could not for bed net use, and for net attitude, the variables were categorized as strongly agree and somewhat agree vs strongly disagree and somewhat disagree, except for the question on “*I do not have time to repair a hole in my net*”. SES was categorized into three levels: lowest SES, middle SES and highest SES. Net care attitude was categorized into two levels: negative attitude and positive attitude. Net coverage indicators, namely (1) Net ownership (proportion of households that own at least one LLIN calculated as number of households surveyed with at least one LLIN divided by the total number of households surveyed), (2) net use (proportion of households that slept under a LLIN the night before the survey, calculated as the number of people that slept under the net the previous night of the survey divided by the total number of people surveyed) and (3) population access (proportion of the population with potential to be protected by an LLIN within their household, assuming each LLIN is used by two people, calculated as number of net used multiplied by 2 divided by the number of people that slept in the household the previous night of the survey) were estimated.

**Fig. 5:**
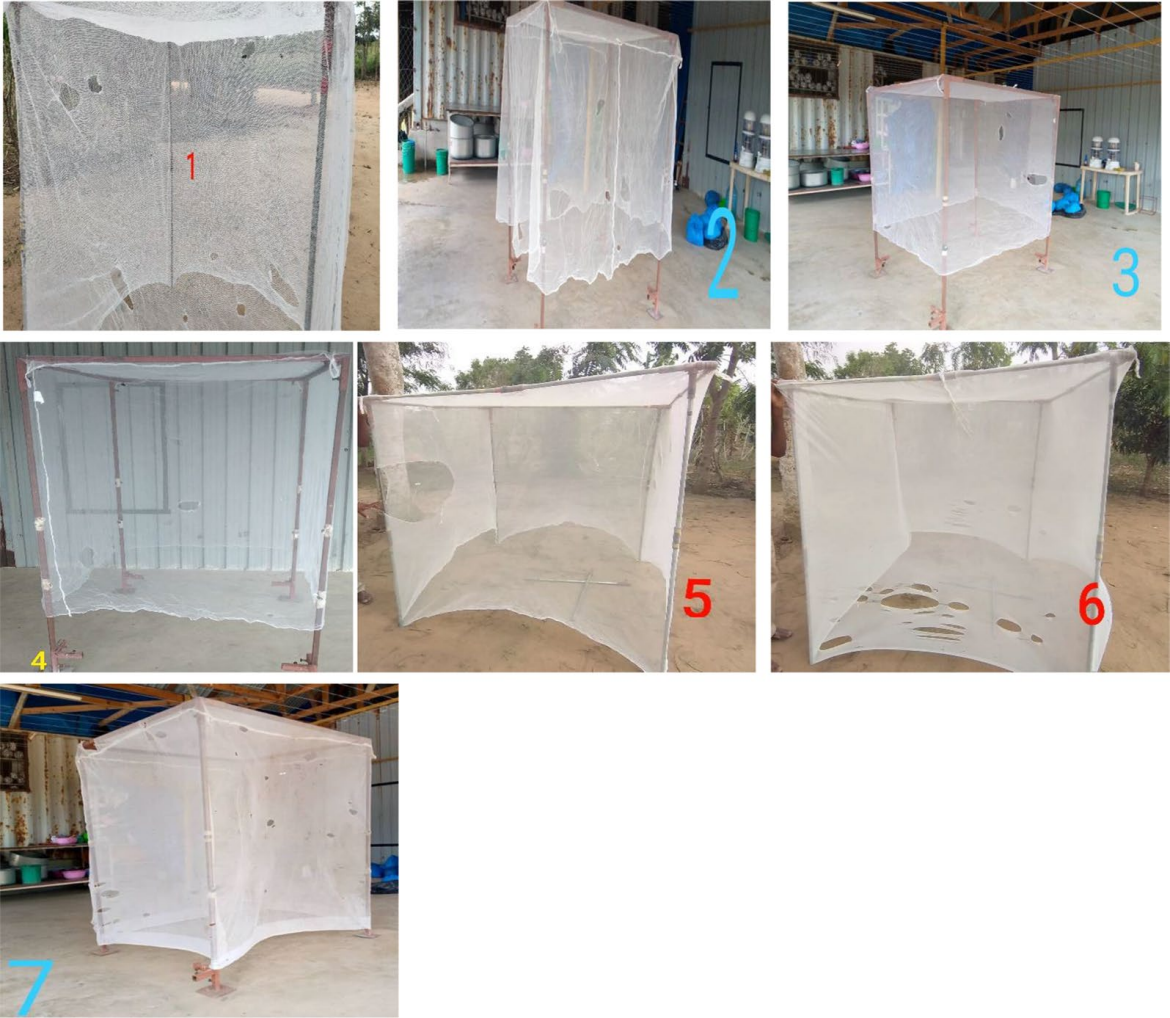
Pictures of all 7 “too torn” nets assessed by respondents

A multivariable binary logistic regression analysis was performed to estimate factors associated with discarding of the WHO “too torn” LLIN assessed. The outcome variable was “bed net ending” which was binary with outcomes “kept” or “discard” as responses for each WHO “too torn” LLIN assessed. The outcome responses from the FGDs were matched with respective data of the individual in structured questionnaire. Primary explanatory factors such as age, sex, level of education, number of occupants per household and SES were considered in the models, in addition to factors that influenced the coefficient by 20% using backward elimination techniques [41]. The too torn LLINs based on WHO pHI calculation, were visually assessed (Fig. 5).

## Results

### Socio-demographic characteristics from the questionnaire

A total of one hundred and seventeen households participated in the FGDs and also responded to the baseline questionnaire of LLIN durability trial. Sixty-nine of the participants were women, and sixty-four of the participants were people of 40–60 years old (55%). A majority (86%) of the participants reported to have primary or higher education. The average size of a household was 4.8 people. Bed net ownership in the study area was 92%. Population access to LLIN was 63%, (95% CI: 56–70%) and 81% (95% CI: 74–87%) of the respondents reported sleeping under a LLIN the night before data collection (Table 2).

**Table 2:** Socio-demographic characteristics of participants

Variables	n (%)
Bed net access	63 (95% CI 56–70%)
Bed net use	81 (95% CI 74–87%)
Age group	
40–60	64 (55)
18–39	53 (45)
Gender	
Men	48 (41)
Women	69 (59)
No formal education	16 (14)
Formal education (Primary-higher)	101 (86)
Household size	
1–5 residents	71 (61)
6 & above residents	46 (39)
Household Socioeconomic Status	
Lowest	37 (33)
Middle	37 (33)
Highest	38 (34)
Study villages	
Kiwangwa	30 (26)
Bago	30 (26)
Msinune	32 (27)
Mwavi	25 (21)
Total (N)	117(100)

### Reasons for discarding nets in the FGDs

**Fig. 6:**
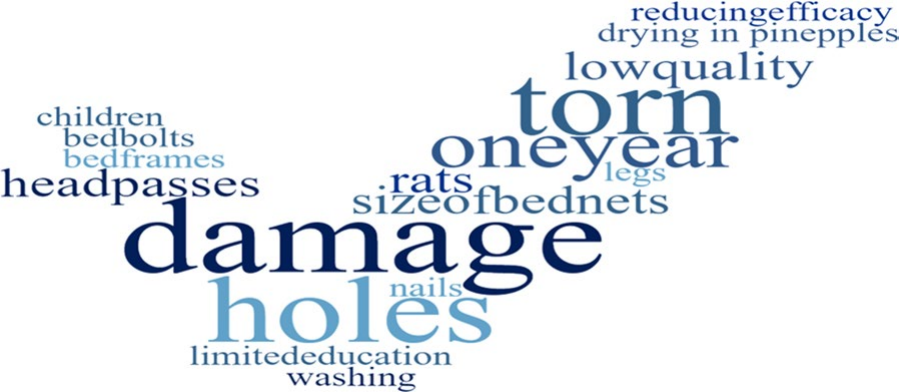
Visual representation (word cloud) of factors associated with discarding of LLINs and reasons for net damage in Bagamoyo, Tanzania

Most of FGD respondents considered physical condition of the LLIN and how it continues to offer protection against mosquitoes as the deciding factor for discarding LLIN. A bed net with many holes or large holes is defined as “too torn” according to participants from the study where by the bed net loses its functional life. LLINs with poor physical condition were discarded even if they were only one or two months old. Participants reported being happy to continue using older nets whose physical condition was good especially when they did not have a new net to replace (Fig. 6). The study found out that the position of the hole on the LLIN can determine whether the LLIN is still useful or should be discarded simply because a hole at the bottom of the LLIN can be tucked underneath the mattress. If holes are in other positions like above the mattress line, the LLIN is more likely to be discarded. However, LLINs with large holes are discarded because the chance that mosquitoes will pass and bite occupants is higher. Participants preferred to use a good LLIN even when it has small holes, they can repair, and continue to use.

*“When the net has large holes that even a person’s head or limbs can pass through, I do not keep that net. But if the holes are small or normal size, I repair the net and keep it.”* (Male, 60).

### Causes of net damage in the FGDs

Respondents were asked what were the causes of damages to the LLIN and how did they protect their LLIN from these damages. The participants answered that damages in LLINs occur due to the following reasons; friction from the mat and edges of the bed, bed bolts, children playing with the LLINs, low quality of LLINs, drying in pineapples/grass, rats, long finger and toe nails, small size of the LLINs compared to the sleeping space, and washing frequency (Fig. 6).

“Damage to the nets is caused by rats, friction from the bed edge or children playing with net. Children can cut holes in the net using a razor/knife” (Female, 23).

### Perceived duration of effectiveness in the FGDs

Most of respondents said that LLINs are effective for about one year. The time in which LLINs remain effective depends on the materials used to make it and the care given to it (Fig. 6). In addition, the participants said that there is a relationship between care for a LLIN and functional life of the LLIN because if a new LLIN is left uncared for, it will not last for a year. When a LLIN is cared for, it can last for years. Furthermore, participants said LLIN obtained from malaria campaigns have to be replaced within a year because they often get old or torn easily after a year.

*“On average, a properly maintained net can last for one year. Even if maintenance is good, it is necessary that the mosquito net should be replaced within one year”* (Male, 45).

### Factors associated with durability in the FGDs

Durability of a LLIN may be influenced by the quality of material used in making it, nature of the house the net is being used, frequency of washing, level of education of head of households and attitude on net care and repair. Additionally, it was noted from the dialogue that the quality of the LLINs motivates people to care about the net. The participants from the FGDs said that the care for the LLIN can determine the durability of a LLIN. The participants indicated the importance of education regarding LLIN care and repair, as many people in the community don’t know how to care and repair a LLIN. Also, it was noted in the group discussions that some people in the communities don’t know how to properly hang bed nets.

“*The issue here is many mosquito nets are of low quality. There was a time I bought a mosquito net and after one month it became too torn. Thus, I replaced it”* (Male, 29).

### General knowledge on bed nets, their use and treatment status in the FGDs

All participants valued LLINs and used them. Respondents knew that sleeping under a LLIN protected them against potentially infective mosquito bites. Also, LLINs offered protection to their family against pests like blackflies, cockroaches, spiders, rats as well as snakes. Participants mentioned that *“in starvation a bone can also be meat*”, it is better to sleep under an old or torn LLIN than without. Additionally, they get a good night sleep (*Wanalala kwa raha*) with no disturbance from mosquitoes when under a LLIN. Yet, majority of participants did not know how to identify insecticidal nets from non-insecticidal (untreated) or to identify when the insecticides are no longer effective. Others perceived the LLINs to be ineffective when mosquitoes could bite them through the LLINs or were able to enter their LLINs.

“*Yes, we are using mosquito nets *because* of many reasons, first is to protect ourselves from mosquitoes. Second, to prevent other vectors, rats, and snakes. Yes, it’s like security*.” (Male, 59).

### Knowledge on net care and repair in the FGDs

Participants differed in opinions on caring and repairing the LLIN. Many of female respondents said that they care for their LLINs. Some younger participants do not care for the LLINs and they do not have time to repair it when it is damaged. They believe that once a LLIN gets physically damaged, its effectiveness against mosquitoes is also lost and will not protect them as it is supposed to do. Therefore, they often replace it no matter how small the hole is. However, the majority of respondents said that caring for the LLINs is very important for LLIN’s integrity. To maximize usage of LLIN, most of participants prevented their LLINs from getting damage by controlling their children, folding or tying up the LLINs during the day and washing it gently.

While the majority of participants cared for the LLINs, only very few had access of information from radio, televisions, and the clinic regarding LLIN care, because the majority of the participants did not own a radio or have access to other media for information. Participants, especially the young women, had inherited the knowledge from their mothers. Therefore, when they grew up and started their family, they did what their mothers taught them about caring and repairing of nets. From the discussions, the participants requested the ministry of health, or other stakeholders to provide education on how to care for the mosquito nets, as it will be very helpful to retain LLINs for a longer period.

“*My opinion is that the ministry *of* health should come to educate us on how to care for the net”* (Male, 28).

### Knowledge on LLIN disposal from the FGDs

The discussions revealed that community were also not informed on proper ways of discarding the old LLINs. There was no communal plan or aware of any guidelines from the local government or instructions that guides on how to dispose the old or torn LLINs. Communities do not know what to do with the old /torn LLINs. Therefore, the lack of guidelines for discarding LLINs is a challenge raised during the discussions. Furthermore, improper discarding of LLINs reported by the groups may lead to environmental hazards such as an unintentional introduction of insecticides to the environments causing pollutions as well as insecticide resistance.

“*Because we have no education (on *LLIN* disposal), everyone uses his/her own preferred approach. When mosquito nets become old or too torn, I do as I please to it. From using it to make a rope to seal charcoal bags to discarding it in the garbage pit*” (Female, 52).

### Methods of discarding old/torn LLINs from the FGDs

**Fig. 7:**
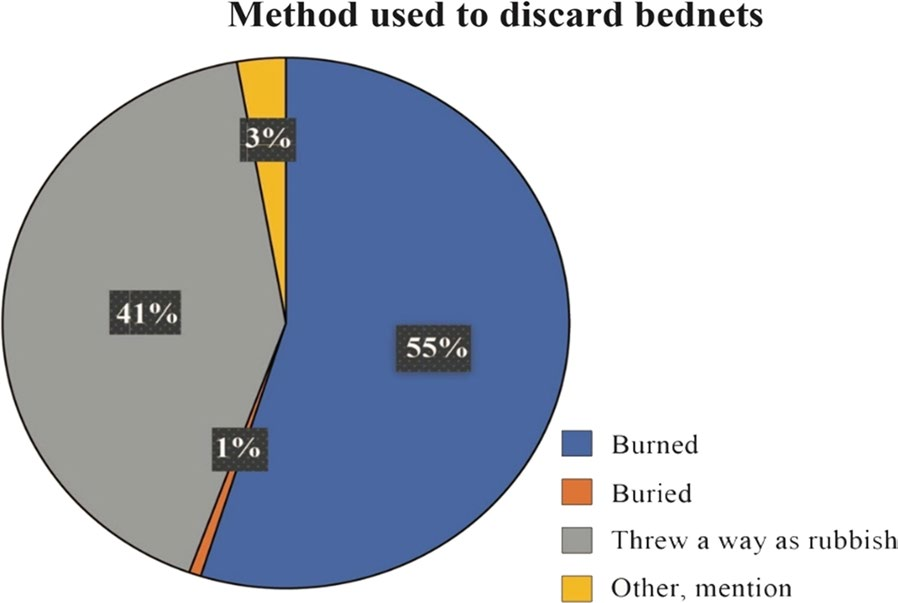
Pie chart of methods used to discard old nets

Responses varied between younger and older participants. Younger participants (18–39 years old*)* often reported that they discarded old LLINs immediately when they received new ones even if the old LLIN had only one hole, while older participants (40–60 years old) stored old LLINs for visitors as well as for future use, and also used LLINs for other purposes when it was perceived to be too torn. Burning or throwing LLINs in a rubbish pit were the common disposal methods of old or torn LLINs (Fig. 7).

### Alternative uses for old LLINs from FGDs

Bed nets were also said to be used for other purposes such as in farming by making garden screen against chicken, making ropes, chicken coops, soccer balls and bags. Others used them as charcoal bags because the material used to make LLIN (polyester and polyethylene) is perceived to be strong and cheap instead of buying charcoal bags from shops, which are considered to be expensive.

” *I usually give old LLINs to my friends *who* sell charcoal because buying rope from the shop which is strong to cover the bags of charcoal is quite expensive”* (Male, 59).”

### Discarding of visually inspected “too torn” nets using participatory data collection in the FGDs

Out of 117 participants of all FGDs, 59% were younger people of 18–39 years old, 65% were male and 64% represented wealth households. These three groups reported the WHO “too-torn” LLINs they inspected to be no longer useful (Table 3). The converse was true with older people, women and less wealthy who were more likely to classify the same nets as “good” instead of “too-torn”. This demonstrated some group differences in how they classified the end of useful life of a LLIN. More than half of all of respondent 55% suggested burning of LLINs as a means of disposal (Fig. 7).

**Table 3:** Percentage distribution of FGDs participants that inspected WHO “too torn” nets and their decision to keep or discard the nets

Variables	WHO too torn condition n (%)			Discard n (%)	
Covariates	Good*	Damaged^‡^	Too torn^†^	Yes	No
Age group					
40–60	185 (41)	72 (16)	191 (43)	192 (43)	256 (57)
18–39	123 (33)	30 (8)	218 (59)	245 (66)	126 (34)
Gender					
Men	90 (27)	26 (8)	220 (65)	240 (71)	96 (29)
Women	218 (45)	76 (16)	189 (39)	197 (41)	286 (59)
Household size					
1–5 residents	190 (38)	67 (14)	240 (48)	250 (50)	247 (50)
6 and above residents	118 (37)	35 (11)	169 (53)	187 (58)	135 (42)
Education					
No formal education	64 (57)	15 (13)	33 (30)	38 (34)	74 (66)
Formal education	244 (35)	87 (12)	376 (53)	399 (56)	308 (44)
Household SES					
Lowest	115 (44)	37 (14)	107 (41)	109 (42)	150 (58)
Middle	105 (41)	36 (13)	118 (46)	125 (48)	134 (52)
Highest	71 (27)	24 (9)	171 (64)	188 (71)	78 (29)
Study villages					
Kiwangwa	57 (27)	51 (24)	102 (49)	120 (57)	90 (43)
Bago	103 (49)	21 (10)	86 (41)	92 (44)	118 (56)
Msinune	95 (43)	12 (5)	117 (52)	121 (54)	103 (46)
Mwavi	53 (31)	18 (10)	104 (59)	104 (59)	71 (41)
Total	308 (38)	102 (12)	409 (50)	437 (53)	382 (47)

* Bed net of a total hole surface area of < 0.001m^2^ (pHI < 64)

^‡^ Bed net of a total surface, ≤ 0.1m^2^ (pHI ≤ 642)

^†^ Bed net of a total surface area of > 0.1 m ^2^(pHI > 642)

In the multivariable analysis, male heads of households were approximately 7 times more likely to discard the WHO “too torn” LLINs that they were shown than their female counterparts [OR = 6.85 (95% CI (4.44 – 10.59), p < 0.001 with overall p =  < 0.001]. Household heads aged 18–39 years had higher odds of discarding “too torn” LLINs [OR = 4.97(95% CI (3.25– 7.32), p < 0.001 with overall p =  < 0.001] compared to older ones.

Socioeconomic status was also associated with discarding of WHO “too torn” LLINs. Households with highest economic status were approximately 4 times more likely to discard “too-torn” LLINs than those from the lowest SES group [OR = 3.88 (95% CI (2.33 – 6.46), p < 0.001 with overall p =  < 0.001]. Materials used in making the LLINs was found to be associated with discarding, where “too-torn “LLINs with a rougher texture (polyethylene) were 11 times more likely to be discarded compared to “too-torn” smoother textured (polyester) LLINs [OR = 11.29 (95% CI (3.39–37.58), p < 0.001 with overall p =  < 0.001]. Dirty “too-torn” LLINs were 4 times more likely to be discarded compared to clean “too-torn” LLINs [OR = 4.13 (95% CI (2.43–7.01), p < 0.001 with overall p =  < 0.001]. For every one unit increase in the number of holes, the odds of discarding WHO “too torn” LLINs increased [OR = 1.05 (95% CI (1.04–1.07), p < 0.001 with overall p =  < 0.001] (Table 4)**.**

**Table 4:** Logistic regression of the factors associated with the discarding of “too-torn” LLINs reported during FGDs in Bagamoyo, Tanzania (N = 117)

Models	Univariable			Multivariable			
Co-variates	OR	95% CI	P-value	OR	95% CI	P-value	Overall P-value
Gender							< 0.001
Women	1			1			
Men	3.63	2.69–4.89	< 0.001	6.85	4.44–10.59	< 0.001	
Age group							< 0.001
40–60	1			1			
18–39	2.69	1.95–3.45	< 0.001	4.97	3.25–7.32	< 0.001	
Education							0.509
No formal education	1			1			
Formal education	2.52	1.66–3.83	< 0.001	1.24	0.65–2.34	0.511	
Household size							0.815
1–5 residents	1			1			
6 & above residents	1.37	1.03–1.82	0.030	1.05	0.70–1.57	0.815	
Household SES							< 0.001
Lowest	1			1			
Middle	1.28	0.91–1.81	0.158	1.62	1.01–2.61	0.047	
Highest	3.32	2.31–4.76	< 0.001	3.88	2.33–6.46	< 0.001	
Study Village							< 0.001
Kiwangwa	1			1			
Bago	0.58	0.39–0.86	0.006	0.26	0.15–0.47	< 0.001	
Msinune	0.88	0.60–1.29	0.513	0.75	0.45–1.26	0.278	
Mwavi	1.09	0.73–1.65	0.651	0.87	0.50–1.50	0.616	
LLIN Material							< 0.001
Smooth	1			1			
Rough	0.15	0.10–0.22	< 0.001	11.29	3.39–37.58	< 0.001	
LLIN cleanliness							< 0.001
Clean	1			1			
Dirty	4.81	3.55–6.52	< 0.001	4.13	2.43–7.01	< 0.001	
LLIN number of holes	1.03	1.02–1.03	< 0.001	1.05	1.04–1.07	< 0.001	< 0.001

### Multivariable analysis of the factors associated with the discarding of WHO “too-torn” LLINs from structured questionnaire data

As was observed in the participatory analysis of FGD responses, the multivariable analysis of questionnaire data also showed that male heads of households were more likely to discard WHO “too torn” LLIN [OR = 8.20 (95% CI (2.48 – 27.14), p = 0.001, with overall p = 0.001] compared to female as well as younger heads of households compared to the older ones [OR = 7.51 (95% CI (2.36 – 23.84), p = 0.001, overall p < 0.001]. Socioeconomic status influenced discarding of LLIN; households with the highest SES were ten times more likely to discard LLINs than those in the lowest SES [OR = 9.66 (95% CI (2.18 – 42.86), p = 0.003, overall p = 0.002]. Having recently repaired their LLINs, having received information on LLIN care and repair, or recalling a family discussion on LLIN care and repair was not associated with a reduction in the likelihood of discarding a “too-torn” LLIN (Table 5). A positive net attitude score was associated with lower likelihood of discarding nets in the univariate analysis but this was no longer significant in the multivariable analysis [OR = 0.38 95% CI (0.15 – 0.97), p = 0.044, overall p = 0.122] (Table 5).

**Table 5:** Logistic regression of factors associated with the discarding of WHO “too-torn” LLINs from participant questionnaire data, in Bagamoyo Tanzania (N = 117)

	Discard nets		Univariate			Multivariable			Overall
	n/N	%	OR	95% CI	P-Value	OR	95% CI	P-Value	P-value
Gender									< 0.001
Women	19/69	39	1			1		0.001	
Men	30/48	61	4.39	1.99–9.64	< 0.001	8.20	2.48–27.14		
Age group									< 0.001
40–60	18/64	37	1			1			
18–39	31/53	63	3.60	1.76–7.89	0.001	7.51	2.36–23.84	0.001	
Education									0.357
No formal education	2/16	4	1			1			
Formal education	47/101	96	6.09	1.32–28.20	0.021	2.56	0.32–20.19	0.372	
Household size									0.251
1–5 residents	29/71	59	1			1			
6 & above residents	20/46	41	1.11	0.53–2.36	0.778	0.50	0.15–1.66	0.259	
Study village									0.068
Kiwangwa	14/30	29	1			1			
Bago	13/30	26	0.87	0.32–2.42	0.795	0.61	0.14–2.59	0.506	
Msinune	7/32	14	0.32	0.11–0.96	0.043	0.23	0.05–1.17	0.076	
Mwavi	15/25	31	1.71	0.59–5.02	0.326	2.22	0.44–11.13	0.333	
Net attitude score									0.122
Negative	14/23	29	1			1			
Positive	35/94	71	0.38	0.15–0.97	0.044	0.36	0.09–1.35	0.130	
Household SES									0.002
Lowest	10/37	21	1			1			
Middle	11/37	23	1.14	0.42–3.14	0.797	1.44	0.36–5.79	0.606	
Highest	27/38	56	6.63	2.42–18.18	< 0.001	9.66	2.18–42.86	0.003	
Repaired nets in the last 6 months									
No	7/14	50	1						
Yes	35/90	83	0.64	0.21–1.97	0.433				
Received information on net use, care & repair									
No	22/56	45	1						
Yes	27/61	55	1.23	0.59–2.56	0.586				
Family discussion on net care & repair									
No	19/49	39	1						
Yes	30/68	61	1.25	0.59–2.63	0.564				

## Discussion

Results from FGDs and LLIN durability trial both show that the physical condition of LLIN, age of head of household, gender and socioeconomic status are the major factors, influencing the discarding of LLIN. The physical condition of a LLIN is very crucial to durability. There was a clear association between LLIN damage and the probability of the LLINs being thrown away. Households reported that they would often use a LLIN until they perceived it to be irreparably damaged. The LLIN was determined expired due to the presence of holes in the LLIN or presence of mosquitoes inside the LLIN. This is consistent with other research, which found that the physical condition of the LLIN and its perceived efficacy is often associated whether the LLIN remains in use or is thrown away [19, 42–44]. The community perceived LLINs to last around one year and that this could be less if there is presence of holes within 2–3 months of use. Durability studies done in Tanzania, Rwanda, Madagascar, Benin and Ethiopia have also reported less than 3 years of durability of LLINs [21, 24, 42, 45–47]. A recent review from forty high malaria burden African countries estimated LLIN durability is around 2 years in Tanzania and even lower in many of the other sub-Saharan countries [4]. Moreover, heads of households mentioned that LLINs offered in malaria campaigns to be of insufficient quality, they get easily damaged after regular use and wash. Therefore, more durable materials may enhance the longevity of LLINs and stakeholders such as National Malaria Control Programmes (NMCP), U.S President’s Malaria Initiative (PMI), Global Fund and manufacturers should consider the resistance to damage [48] of LLINs procured to maximize their longevity and reward the manufacture of good quality products. This finding is consistent with a cross-sectional study in Ghana that showed that householders were willingly to pay for better LLINs [49]. Householder's perceptions of LLINs were clearly related to the physical characteristics of the LLINs with dirty LLINs and rough (polyester LLINs) were more likely to be discarded when damaged in this setting. User preference for polyester LLINs has been seen in other studies as they are softer to the touch: Tanzania [50], India and Nepal [51] Madagascar [52] and Vietnam [53].

It has been found in the discussion that older people with many people within household keep bed nets the longest. It is probably that high density households are more likely to attract mosquitoes. The most likely option for the household is therefore to retain bed nets as long as possible to protect household occupants. Households headed by women were more likely to keep damaged LLINs because in general households headed by women are more likely to be poor [54, 55] and, therefore, often do not discard LLINs as it costs money to change. Women keep damaged LLINs for future uses like when they have visitors. Behavioural point of view about the use of bed nets was that it is better to sleep under a damaged LLIN than without a mosquito net [25, 56]. LLIN retention is also important as when coverage is incomplete, school children are often left without a LLIN [57] due to within household prioritization and allocation of sleeping spaces [58]. These children bear a great burden of malaria at a critical life stage and have long been reported to be the group that contributes much of the ongoing malaria transmission [59–64].

Those who have more income were less tolerant to damaged LLINs. Households with more income can afford to buy LLINs and hence replace their free programme nets [58, 65]. The association between poorest wealth quantile and determinants of damages was due to poor house structure, crowding, and absence of adequate sleeping places [9, 44, 66]. Therefore, those in the poorest group were most likely to have damaged LLINs, but more likely to hold on to them in the absence of a replacement unless their sleep is disturbed [65]. Although, it is better for those without access to newer bed nets to continue using the old/torn net until they get new LLIN or as long as the old bed nets is providing some protections against mosquitoes as recommended by the WHO [27]. However, the premature discarding of torn LLINs that are still insecticidal by the wealthier is a concern if LLINs are poorly discarded in the environment also considering that access to nets is low. Thus, BCC should encourage gifting of LLINs to those without access in this group and inform community to discard LLINs especially those, which are too torn and lost their insecticidal durability as recommended by the WHO.

### Alternative use of old LLINs

This study found that most LLINs were thrown away or burned and a few of too-torn LLINs are used for other purposes once they are perceived to be non-functional against mosquitoes. Alternative purposes of LLINs found in this study are also reported in Kenya, Malawi, Ghana, Senegal, Nigeria and Uganda [18, 67–69]. In addition, due to the perceived strength of the material used to make LLINs, they are sold as ropes or bags thereby an alternative source to generate income. The participants pointed out that they used LLINs for the aforementioned alternative purposes, because the standard materials such as sisal ropes were more expensive to purchase directly from the shops. LLINs made of stronger fibers (polyester and polyethylene) offered a cheaper alternative; of which the LLINs received from the mass campaigns in the community was Olyset LLIN. Previous studies done by Randriamaherijaona et al. [46] and Allan et al. [70] found that polyethylene (Olyset LLINs) were more durable and accumulated less damage than polyester LLINs (PermaNet). More recent research has found that polyethylene (Olyset) LLINs are more prone to damage than polyester (PermaNet) LLINs [24, 71, 72]. No one repurposed old LLINs for continued malaria control such as house screening or to close eave gaps through which mosquitoes enter even though this can be easily done and may offer substantial relief from mosquito bites [73]. This could be due to lack of information/education that these methods could offer continued protection against malaria mosquitoes. Screening windows and eaves with netting has been observed to decrease mosquito entry in multiple studies [74–77]. Findings from this study shown that, people in the community do not follow current recommendations for beneficial repurposing once an LLIN is no longer useful or old. They discard LLIN when it may still be useful as a barrier for mosquitoes like curtains, patching wall holes and viable nets, eaves and constructing window or door screening for protection against malaria infection. Although, some of the study participants said that they do not have knowledge on alternative uses of bed nets.

### Premature discarding of LLINs

This study observed that young people prematurely discarded LLINs because of small holes that could have been repaired as noted by the older people. The older people repaired holes in LLINs by sewing, tying, stitching or tacking. LLIN repair extends LLIN lifespan, which is crucial for protection. Therefore, it is necessary to emphasize and improve behaviour change communication (BCC) to promote net retention. BCC refers to the strategic use of communication approaches to promote changes in knowledge, attitudes, norms, beliefs, and behaviour [32]. Therefore, BCC can be used among young people as well as community to encourage LLIN care to prevent quick deterioration and promote retainment especially if the LLIN was acquired recently. While malaria prevention is taught as part of the curriculum in all Tanzanian schools, LLIN care and repair is not. Extending the curriculum to include LLIN care could be an important means of encouraging young people to value and care for their LLINs.

### Perception of WHO “too torn” LLINs as “good”

Our study showed that participants who were older were more likely to classify an LLIN in WHO “too-torn” category, i.e. unlikely to provide physical protection against mosquito bites as “good” or “damaged”. The same was for women, and those from low SES households and also the ones who were less educated. The findings are not surprising because in the study area older people have the culture of keeping household utensils and materials, including nets for long compared to the young ones. Women are more likely to be responsible for the care of nets used in the household than men, the attitude of taking care of the nets might have influenced their decision to keep a too-torn net with the mindset of repairing it afterwards. It is known that wealthy households do not usually rely solely on mass campaigns to have a new bed net unlike their counterparts [78]. Households with low SES tend to keep too-torn nets and repair them it until they obtain a new net, as mentioned in the study that having a too-torn net is better than none.

### Methods used for discarding of nets

Although majority of the respondents reported improper discarding of LLINs such as discarding in the public rubbish pit and burning, however this is not recommended because it has negative environmental impact. Research done by Kudom et al. in Ghana reported high level of pyrethroid resistance of *Anopheles* species in an urban setting without urban agriculture which was postulated to be due to improper discarding of old insecticides such as LLINs, domestic insecticides as well as the use of herbicides [79]. Agricultural areas where they intensively use insecticides have observed resistance of pyrethroids of vectors that are important to public health [80, 81]. In addition, burning of LLINs is very damaging to the environment and human health [82], also disposal should be buried away from water bodies to protect aquatic organisms as well [27]. Respondents felt that they did not have enough information on the correct disposal of LLINs and this could be overcome by adding this information to the LLIN label or packaging. Additionally, NMCP and other stakeholders could work together with the ministry of environment to make local regulation and follow WHO guideline for discarding of LLINs so as to protect environment and improve peoples’ health [27].

### Care for the LLINs

Study participants reported that they care for their LLINs by washing, repairing and tying although LLINs in other studies in Tanzania were not often found to actually be repaired [66, 83]. Consistent care for the LLIN was also reported by other studies done in Senegal [84], Nigeria [85], Ethiopia [86] and Kenya [68]. In practice, LLINs are cared for by washing, tying up during the day, sometimes drying in the shade but rarely repaired. Participants mentioned a desire to receive messages on the importance of care and repair, which could motivate repair and increase LLINs lifespans. Older male respondents said that the cost of getting treatment from the health facility when a child is sick from malaria are usually high, so it is better to care for their LLINs making them functional and protective for a long time to minimize hospital associated costs. This study observed that younger people were not readily caring for their LLINs because they are preoccupied with youth activities and schedules of their own. Very few respondents reported to have received information on LLIN care and repair from the radio and/or television because they lack of such devices, about 48% owned radios and 23% owned television. This was also seen in Ethiopia where < 25% of respondents owned a radio which led few people to receive the intended messages [87]. Interestingly, in this study, women reported to get the knowledge from their mothers. Women are primarily the ones who take care and repair LLINs in households [22, 88]. While there was low level of knowledge on LLIN care and repair participants are interested in learning how to care for their LLINs to make them longer lasting. Targeting this kind of information to younger age groups could be useful to ensure continuity across generations as was observed by the younger women who credited their mothers for the information.

Concern was raised on receiving information via radio. The younger population rarely listens to the radios and when they do, they often listen to the music and ignore other programmes. Therefore, BCC should reach out to music artists for promotion of LLIN care and disposal educational information as well as through schools and health facilities. Government and other stakeholders should consider providing training during LLINs distribution to improve life span of LLINs and to reduce misuse of LLINs and premature discarding of LLINs. National Environment Management Council (NEMC) of Tanzania may provide guidance on disposal of old/torn LLINs, or other acceptable alternative uses like curtains for doors and windows as a means of forming a barrier against mosquitoes and vectors of other infectious diseases, to eliminate improper discarding of LLINs and protect environment pollution. Bundling information on all the areas where participants expressed interest in learning more, i.e. proper use, care and disposal of LLINs may be best bundled with LLINs at distribution either on packaging, on the label or with the LLIN on a leaflet.

The study has a number of limitations. The FGDs were conducted among a group of people that had already taken part in a LLIN durability study. Therefore, they had been recently sensitized on use and importance of LLINs, thus, may have a higher than an average knowledge of LLINs’ use. The color of LLINs distributed by the durability study was white which is not preferred by the study participants and may have affected their decision to discard the nets. In addition, the LLINs shown to participants were not of a wide range of damage levels, all LLINs were too torn based on WHO pHI, and it would have been better if a wider range of LLIN damage levels that could be considered as “good” and “serviceable” were made available. It is also likely that the participants’ responses were biased to a certain extent by peer pressure as many reported repairing their LLINs.

The study interviewed only those who discarded bed nets, this may have introduced some bias in the data therefore, opinions gathered may be one sided despite intensive probing on discarding and retaining of bed nets practices in the communities.

## Conclusion

Factors associated with discarding of LLINs were poor LLIN physical condition, socioeconomic status, age of head of household and gender. Government and other stakeholders should consider a more robust LLINs that resist damage for distribution in the community to increase the longevity of LLINs. BBC could be implemented during LLINs distribution to improve life span of LLINs by sensitizing users on misuse of LLINs, and premature discarding of LLINs. Means of providing BCC appropriate to this context are information provided with LLINs at delivery (on bags, labels or a leaflet), messaging by music artists and information via the school curriculum for promotion of LLIN care and disposal educational information. Furthermore, Government Ministry of Health, NMCP, and other stakeholders should consider reducing time period of distributing LLIN below three years because LLIN are not staying three years of functional life in the field.

## Supplementary Information

**Additional file 1: Table S1.** Bed net use questions. **Table S2.** Net attitude questions.

## Data Availability

The datasets used during the study are available from the corresponding author on reasonable request.

## Abbreviations

LLINs: Long-lasting insecticidal nets
FGD: Focus group discussion
WHO: World Health Organization
SES: Socioeconomic status
CI: Confidence interval
PCA: Principal component analysis
IRB: Institutional review board
NIMR: National Institute for Medical Research
HH: Head of household
BCC: Behavioural change communication
NMCP: National Malaria Control Programme
PMI: U.S President’s Malaria Initiative
USAID: United States Agency for International Development
SNP: School net programme
NEMC: National Environment Management Council
