# Supplementary material for: “In starvation, a bone can also be meat”: a mixed methods evaluation of factors associated with discarding of long-lasting insecticidal nets in Bagamoyo, Tanzania
Source: Malar J. 2022 Mar 24;21:101. doi: 10.1186/s12936-022-04126-5 (PMC8944021; doi:10.1186/s12936-022-04126-5)
Supplement: Supplementary file 1 — Additional file 1: Table S1. Bed net use questions. Table S2. Net attitude questions. [file 12936_2022_4126_MOESM1_ESM.docx]

**SOM Table 1: Bed net use questions.**

|  | Variables | Definitely could | Probably could | Probably could not | Definitely could not |
| --- | --- | --- | --- | --- | --- |
| Q1 | Obtain enough bed nets for all your children. | 1 | 2 | 3 | 4 |
| Q2 | Hang a bed net above your children’s sleeping spaces. | 1 | 2 | 3 | 4 |
| Q3 | Protect yourself and your children from getting malaria. | 1 | 2 | 3 | 4 |
| Q4 | Save enough money to obtain bed nets for all your children. | 1 | 2 | 3 | 4 |
| Q5 | Sleep under a bed net every night of the year. | 1 | 2 | 3 | 4 |
| Q6 | Get all of your children to sleep under a bed net every night of the year. | 1 | 2 | 3 | 4 |

**SOM Table 2: Net attitude questions**

| Number | Variables | Strongly agree | Somewhat agree | Somewhat disagree | Strongly disagree |
| --- | --- | --- | --- | --- | --- |
| Q1 | Mosquito nets are valuable | 1 | 2 | 3 | 4 |
| Q2 | There are actions I can take to make my net last long | 1 | 2 | 3 | 4 |
| Q3 | It is not possible to repair holes in nets | 1 | 2 | 3 | 4 |
| Q4 | A repaired net can still be effective against mosquitoes | 1 | 2 | 3 | 4 |
| Q5 | Other people in this community fix holes in their mosquito nets | 1 | 2 | 3 | 4 |
| Q6 | I do not have time to repair a hole in my net | 1 | 2 | 3 | 4 |
| Q7 | I can help protect my family from malaria by taking care of my net | 1 | 2 | 3 | 4 |
| Q8 | I am confident I can repair holes immediately | 1 | 2 | 3 | 4 |
